# Evaluation of ballistics euthanasia applied to stranded cetaceans using ethological and post-mortem computed tomography assessment

**DOI:** 10.1007/s11259-024-10537-3

**Published:** 2024-09-17

**Authors:** Rebecca M. Boys, Brian C. W. Kot, Gordon Lye, Ngaio J. Beausoleil, Stuart Hunter, Karen A. Stockin

**Affiliations:** 1 Cetacean Ecology Research Group, College of Sciences, Massey University, Private Bag 102-904, Auckland, New Zealand; 2 Department of Infectious Diseases and Public Health, Jockey Club College of Veterinary Medicine and Life Sciences, City University of Hong Kong, Hong Kong, China; 3 Animal Referral Centre, 224 Albany Highway, Schnapper Rock, Auckland, 0632 New Zealand; 4 Animal Welfare Science and Bioethics Centre, School of Veterinary Science, College of Sciences, Massey University, Private Bag 11-222, Palmerston North, New Zealand; 5 School of Veterinary Science, College of Sciences, Massey University, Private Bag 11-222, Palmerston North, New Zealand

**Keywords:** Behaviour, Euthanise, Insensibility, Odontocete, Stranding, Welfare

## Abstract

Debilitated stranded cetaceans with low survival likelihood, may require euthanasia to avoid further suffering. Euthanasia can involve chemical or physical methods, including ballistics. Ballistics should cause instantaneous, permanent insensibility through brainstem disruption. Despite wide application, there is limited understanding of ballistics-related welfare outcomes. We opportunistically examined behaviour of three maternally-dependent cetaceans following shooting and the related cranial disruption post-mortem using computed tomography (PMCT). Our aim was to understand whether a ‘humane death’, i.e., euthanasia, was achieved. Each animal was shot using different projectile types: soft non-bonded, solid, and soft bonded. In two animals, insensibility was not immediately assessed following shooting, although both were reported as ‘instantaneously insensible’. From our analysis, all animals displayed musculoskeletal responses to shooting, including peduncle stiffening and slack lower jaw, followed by musculature relaxation 24-, 10.3- and 20.8-seconds post-ballistics, respectively. The animal shot with a soft non-bonded projectile also displayed agonal convulsions and tail-lifting for 16-seconds post-shot; these were not observed for solid or soft bonded projectiles. PMCT findings indicated projectile disruption to the brainstem and/or spinal cord likely to cause near-instantaneous insensibility. However, extra-cranial wounding was also evident for the soft non-bonded projectile, highlighting potential for additional welfare compromise. Our results demonstrate that ballistics can achieve a relatively rapid death in young, stranded cetaceans, but careful equipment selection is required. To ensure a humane death, verification of insensibility must be undertaken *immediately* following shooting. Further studies should be undertaken to improve knowledge of appropriate procedures and equipment for euthanasia, ensuring humane deaths for compromised cetaceans.

## Background

Stranded cetaceans are often found in a debilitated condition (Arbelo et al. 2013; Herráez et al. 2013; Diaz-Delgado et al. 2018; Câmara et al. 2020) and end-of-life decisions, including euthanasia, may be required to prevent further welfare compromise (humane ending of life for an animal that is otherwise suffering (Boys et al. 2021). Methods for the euthanasia of cetaceans include chemical and physical techniques, yet there are limited data available to inform development of standardised approaches (Barco et al. 2016; Boys et al. 2021; Stringfellow et al. 2022). In some regions, including Aotearoa New Zealand, chemical euthanasia may not be possible due to a lack of veterinary personnel to administer the euthanising agent(s) and/or due to concerns about eco-toxicity, and human safety considerations (O’Rourke 2002; Bischoff et al. 2011). Instead, physical methods, such as ballistics, are applied.

To be considered euthanasia, the death of the animal must be achieved in such a way as to minimize negative impacts on its welfare (i.e., be humane). In the context of ballistics, key structures in the brain must be destroyed rapidly to ensure that the animal is rendered irreversibly unconscious i.e., insensible (Greer et al. 2001). This should include destruction of the brainstem (midbrain, pons and medulla oblongata) (Leary et al. 2020) which controls respiratory and cardiac function (Millar and Mills 2000; Cozzi et al. 2016; DeNicola et al. 2019; Panneton and Gan 2020) and is key to the generation of conscious awareness though the reticular activating system (Parvizi and Damasio 2003; Grady et al. 2022; Taran et al. 2023).

However, the application of ballistics to cetacean species can be complex due to the integument and thick muscle on the parietal and occipital areas of the skull and the dense bones of the cranium (Harms et al. 2018; Roston and Roth 2019). Furthermore, there is high variability in skull morphology and vertebral topography among cetacean species and age groups which, along with the lack of species-specific, detailed protocols, can lead to inaccurate projectile placement and/or the use of inappropriate equipment (Boys et al. 2021, 2022a, 2023). If a cetacean is not rendered immediately unconscious following application of ballistics, it may suffer severely (IWC 2014). Accordingly, it is vital that euthanasia procedures are routinely reviewed to better understand the efficacy of the method and to highlight any potential welfare concerns.

The relative humaneness of killing can be evaluated by assessing the duration and intensity of any negative impacts (suffering) that occur prior to the animal becoming permanently insensible (Littin et al. 2004; Leary et al. 2020). Duration can be quantified through parameters such as the time from the application of the method until the time at which the animal can be verified as insensible or dead (Knudsen 2005; Gales et al. 2008). Examination of behavioural and physiological variables can be used to infer potential suffering (Beausoleil et al. 2016). Additionally post-mortem examination of the resulting penetrating head injuries and relevant lesions can be assessed to infer both the duration and intensity of negative impacts experienced by the animal (Millar and Mills 2000; Thomson et al. 2013; Schiffer et al. 2014; DeNicola et al. 2019; Lund et al. 2021; Boys et al. 2023). Computed tomography (CT) is one of several tools that can be used in post-mortem examinations and provides a non-invasive method to examine lesions (Tsui et al. 2020; Kot et al. 2022); this method has been used to delineate the trajectory, penetration and impacts of projectiles in animals (Millar and Mills 2000; Thomson et al. 2013; Schiffer et al. 2014).

New Zealand is world renowned for its frequent live cetacean stranding events. Two of the most common species to strand in this region are long-finned pilot whales (*Globicephala melas edwardii*) and pygmy sperm whales (*Kogia breviceps*) (Betty 2019; Plön et al. 2023). Due to the low survival likelihood and negative welfare impacts, dependent animals found stranded alone or with deceased mothers are considered non-viable for rescue in New Zealand, as stated in the Department of Conservation Te Papa Atawhai (DOC) Standard Operating Procedures (Boren 2012; Boys et al. 2022a). Therefore, neonates and non-weaned calves are an age class to which killing methods (i.e., ballistics) are mostly likely to be applied. Herein, we present three case studies in which we evaluate the application of ballistics as a method to humanely kill stranded dependent odontocetes (long-finned pilot whale, pygmy sperm whale and a killer whale (*Orcinus orca*).

To make inferences about the welfare impacts of the ballistics method and determine whether the application of ballistics in these cases can be classed as ‘euthanasia’, we describe animal behavioural responses and post-mortem lesions. Specifically, we examine (1) the application of ballistics and the animal’s behavioural responses to the procedure; and (2) findings of penetrating head injuries using post-mortem computed tomography (PMCT). In particular, we utilise PMCT to assess the ballistics impacts including projectile entry and exit sites, intracranial fragments, projectile tracks and their relationship to brain regions and skull-based structures, pneumocephalus, opening of the ventricles, effacement of the basal cisterns, brain herniation and mass effect. Finally, we interpret the disruption to the cranial structures, particularly the brainstem, achieved via ballistics to infer the likelihood that the method caused instantaneous insensibility (irreversible unconsciousness) and highlight potential welfare implications.

## Case presentation

### Animals included in this study

No animals were euthanised for the purpose of this study. All data were opportunistically collected from stranding events where the animal was deemed to require euthanasia by DOC (legislative agency responsible for managing strandings) in partnership with iwi (local indigenous Māori).

#### Long-finned pilot whale (*Globicephala melas edwardii*)

**Fig. 1:**
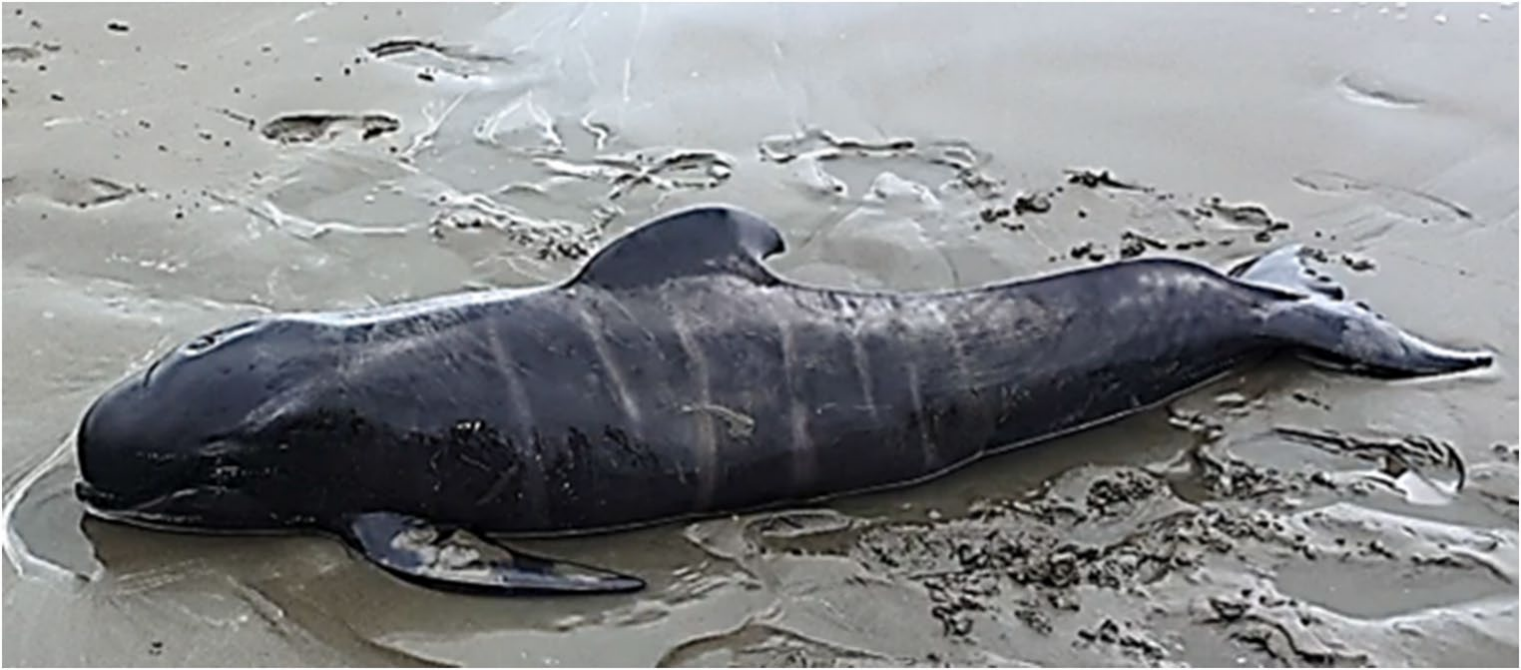
Maternally-dependent long-finned pilot whale (*Globicephala melas edwardii*) that stranded on South Island, New Zealand. Note: evident foetal folds and curved dorsal fin indicating newborn status. Photo credit: Department of Conservation Te Papa Atawhai New Zealand

In 2021, a 1.9 m pilot whale was observed live-stranded on a South Island beach in New Zealand. It is unknown how long the animal had been stranded. Despite the recommendation in the New Zealand DOC SOP relating to animals of this age class, two unsuccessful re-floatation attempts were undertaken by members of the public. Due to the evident maternal dependency (apparent by size and foetal folds; Fig. 1), and with no sign of a natal pod, the decision to euthanise was made by DOC in collaboration with iwi.

#### Pygmy sperm whale (*Kogia breviceps*)

**Fig. 2:**
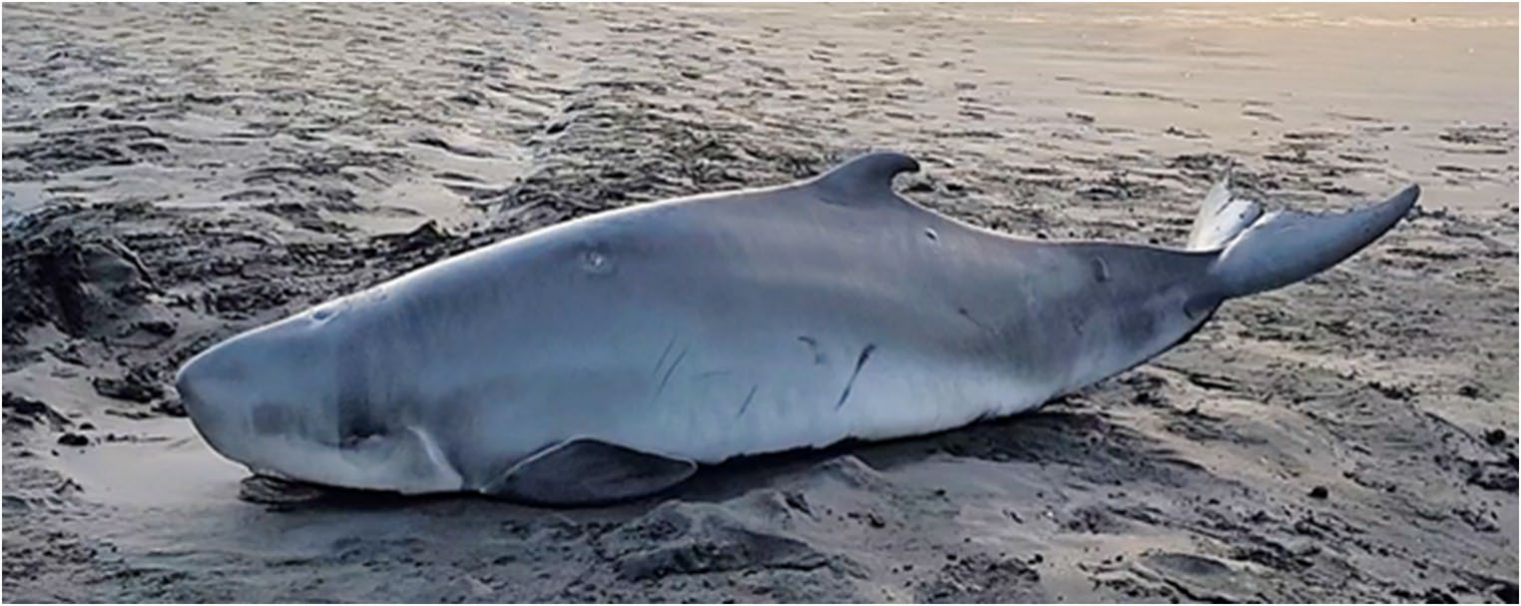
Maternally-dependent pygmy sperm whale (*Kogia breviceps*) that live-stranded with a deceased adult, presumed to be its mother, on North Island, New Zealand. Photo credit: Department of Conservation Te Papa Atawhai New Zealand

In 2023, a 1.63 m pygmy sperm whale (Fig. 2) was reported live-stranded on a North Island beach in New Zealand. The presumed mother was also observed stranded though already deceased. It is unknown how long the animals had been stranded. No re-floatation attempts were undertaken, as total body length indicated likely maternal dependency. Therefore, the decision to euthanise was made by DOC in collaboration with iwi.

#### Killer whale (*Orcinus orca*)

**Fig. 3:**
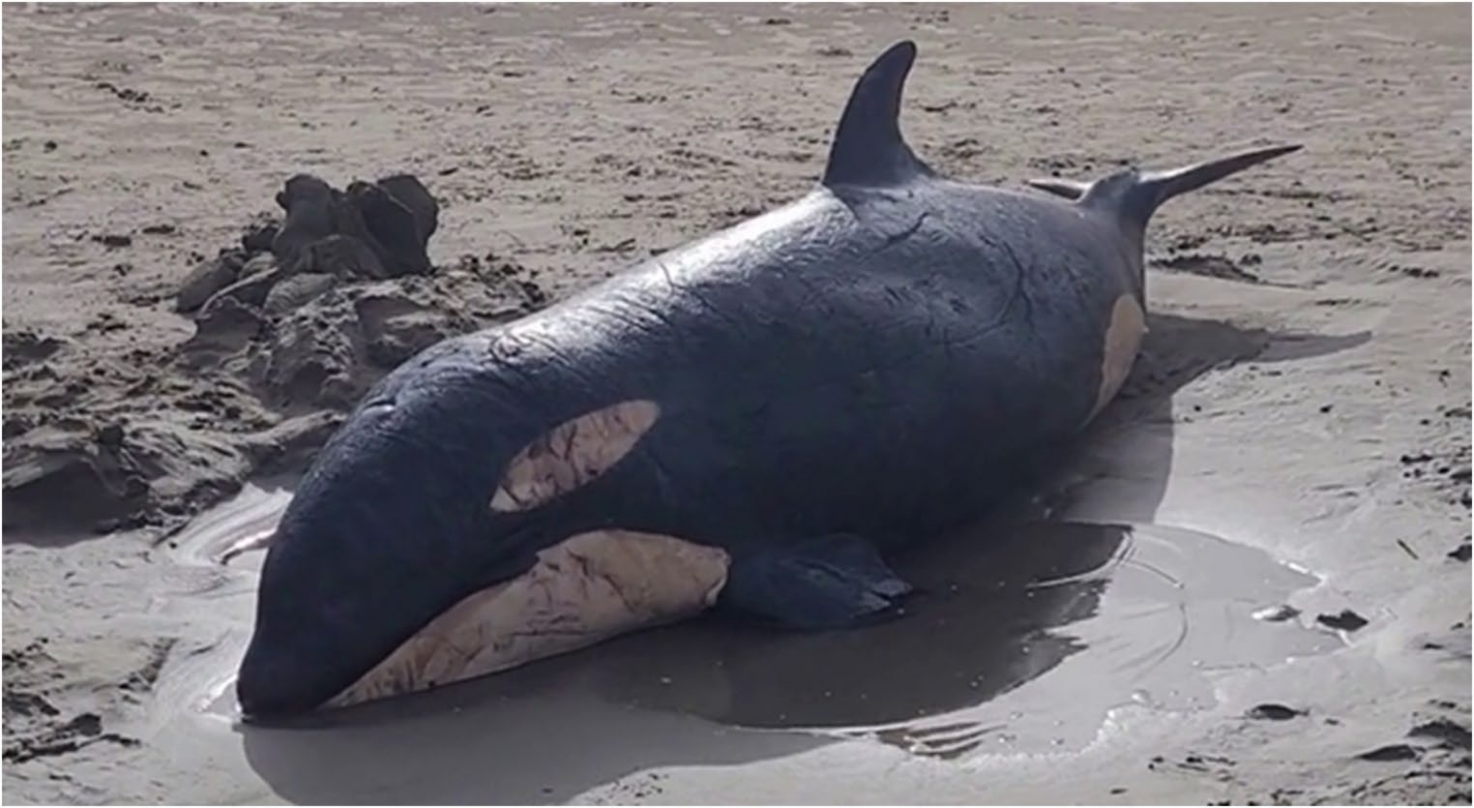
Maternally-dependent killer whale (*Orcinus orca*) that live-stranded on North Island, New Zealand. Photo credit: Department of Conservation Te Papa Atawhai New Zealand

In 2024, a 2.8 m killer whale (Fig. 3) was reported live-stranded at a beach on the North Island, New Zealand. It is unknown how long the animal had been stranded. No re-floatation attempts were undertaken, as total body length indicated likely maternal dependency. Therefore, the decision to euthanise was made by DOC in collaboration with iwi.

### Behavioural responses

Filming of each animal before, during and immediately following the application of ballistics was conducted to enable an examination of the procedure and the animal’s behavioural responses, following Boys et al. (2023). Video footage was taken continuously on a handheld mobile phone camera positioned approximately 2 m in front and to the side of the animal. All available video footage was examined at 0.5x speed using behaviour analysis program BORIS (Friard and Gamba 2016) to assess: orientation of firearm discharge, approximate projectile entry location based on animal anatomical features, number of shots applied, and fine-scale animal behaviour during- and post-application of ballistics. Where possible, time to insensibility was estimated from the video as the time taken from the first shot until absence of reflex responses when tested by the marksperson in the field. In cases where reflex testing was not undertaken, time to insensibility could not be estimated.

Animal behaviours and related human activities were characterised following the ethograms in Boys et al. (2022b, 2023) with new behaviours characterised if observed. All behaviours were coded per second and the frequency (point behaviours), or duration (state behaviours) determined. The pattern of behaviour over the observation period was mapped graphically and described. Due to the stochastic nature of strandings, the duration filmed for each animal varied: the long-finned pilot whale was filmed for 154.8 s before and 88.7 s post-application of ballistics, the pygmy sperm whale was filmed for 27.6 s before and 183.1 s post-application of ballistics, and the killer whale was filmed for 66.3 s before and 210.3 s post-application of ballistics.

### Follow-up procedural data collection

Within seven days of the stranding event, an email was sent to the marksperson involved in the euthanasia procedure with a set of questions to gather additional information about the procedure. This included: firearm type and projectile characteristics, anatomical landmarks used when aiming, entrance point and angle of penetration, whether (from their point of view) insensibility/death criteria were assessed and if so, what criteria were assessed, how assessments were made, and their estimation of time to insensibility/death. Markspersons were also able to provide any additional comments or contextual information they wished. This information was integrated with the data from the video footage to provide further context to the ballistics procedure and explore any potential animal welfare concerns.

### PMCT examination

The pilot whale carcass was carefully transported to a South Island radiological facility for PMCT scan immediately (< 1 h) following ballistics application. The pygmy sperm whale carcass was transported to the cetacean pathology unit at Massey University, Auckland where it was stored at 4 °C for < 24 h at which point it underwent PMCT scanning at a second radiological facility in North Island. Due to the cultural requirement for immediate burial following scanning or other cultural restrictions, a full post-mortem examination to explore ballistics-related injuries could not be undertaken on either carcass. The killer whale carcass was transported to the veterinary pathology facility at Massey University, Palmerston North where it was stored at 4 °C for < 72 h at which point it underwent PMCT scanning and subsequent necropsy following standard protocols (IJsseldijk et al. 2019).

All PMCT scans followed the standardized methods outlined in Kot et al. (2020b) and Tsui et al. (2020), utilising full body scans where possible, with analysis focusing on ballistics related injuries in the head, neck, and thorax regions. CT scanning was optimised to minimise metallic artefacts and an extended CT scale was used to view these artefacts (Link et al. 2000; Fraga-Manteiga et al. 2014). The pilot whale PMCT was undertaken on a Siemens multi-slice spiral CT scanner SOMATOM Drive (Siemens, Healthineers, Germany) unit at 140 kV and 280 mAs with slice thickness of 0.60 mm. The pygmy sperm whale PMCT was undertaken on a Siemens multi-slice spiral CT scanner SOMATOM go.Up (Siemens, Healthineers, Germany) unit at 130 kV and 185 mAs with a slice thickness of 0.70 mm. The killer whale PMCT included scans of only the cranial region, scanning was undertaken on a Philips Big Bore multi-slice helical spiral CT scanner (Philips, Amsterdam, Netherlands) unit at 120 kV and 333 mAs, with a slice thickness of 0.8 mm. In all cases, bone and soft tissue kernel reconstructions were acquired for interpretation of the ballistics-related injuries. CT images were reviewed using commercially available DICOM viewing software (Medixant. RadiAnt DICOM Viewer and Intelerad Medical Systems Incorporated, InteleViewer). Multiplanar reconstruction (MPR) was used to generate transverse, sagittal and dorsal plane reconstructions. The images were displayed in reconstructed windows with adjustments to windowing and levelling as needed during interpretation.

All PMCT data were reconstructed and interpreted by a diagnostic imaging researcher (BCWK) and a final-year veterinary diagnostic imaging resident (GL). Analysis focused on evaluating ballistics-related soft tissue findings (brain oedema, galea hematoma, temporal muscle haemorrhage, subarachnoid haemorrhage, subdural haemorrhage, epidural haemorrhage, intracerebral haemorrhage, ventricular haemorrhage), projectile-related findings (projectile entry, projectile path, projectile exit, retained projectile, projectile fragments, metallic artefacts), and bone findings (fracture lines, fracture fragments, intracranial bone displacement). Furthermore, we also utilised the PMCT to examine for wider welfare-relevant features (e.g., air embolism, atelectasis, blood aspiration, blood loss) related to the stranding.

**Fig. 4:**
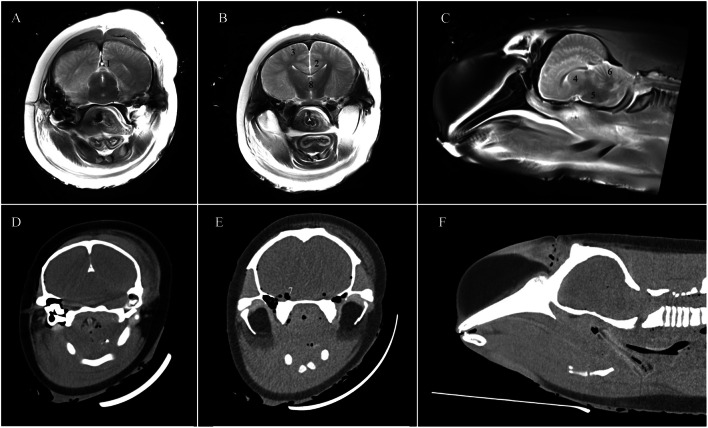
T2 weighted post-mortem magnetic resonance (**A**–**C**) and post-mortem computed tomography (**D**–**F**) images illustrating regions of the cetacean brain: (1) dorsal sagittal sinus; (2) ventricles; (3) grey and white matter junction; (4) basal ganglia; (5) brain stem; (6) cerebellum; (7) intracranial gas accumulation; (8) brain midline/symmetry (Figure from Kot et al. 2020b)

Since dissection of all animals was not possible and damage to neuronal tissue cannot be robustly evaluated from PMCT (Kot et al. 2020b), we defined efficacy of the method (likelihood of causing instantaneous insensibility) based on direct physical disruption to key structures including the brainstem, spinal cord, C1 and occipital condyles (Fig. 4).

###  Long-finned pilot whale: behavioural responses to ballistics application

**Fig. 5:**
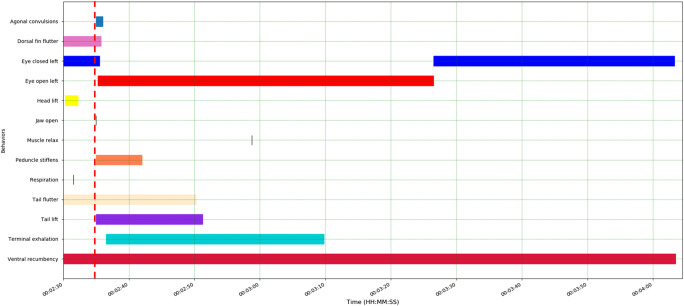
Behavioural events relating to the application of ballistics to the long-finned pilot whale (*Globicephala melas edwardii*). The shot is indicated by the red dashed line. Total recording/observation duration: 244 s; ~95 s relevant to ballistics application shown here

Ballistics was undertaken using a 0.308 calibre rifle with one soft non-bonded 155 gr boat-tail hollow point projectile. The single firearm discharge occurred with the marksperson standing in front of the animal, with the firearm muzzle positioned approximately 30 cm above and in front of the animal’s head, angled approximately 70° ventro-caudally to the right of the animal’s dorsal midline, aiming just caudal to the blowhole. The shot entered caudal and slightly to the right of the animal’s blowhole. Notably, based on the video footage, criteria for verifying insensibility were not assessed in the field following the application of ballistics. A total of 10 animal behavioural responses were characterised from the video footage following firearm discharge (Fig. 5).

Following the shot, stiffening of the peduncle occurred immediately (< 1 s) and remained in this tonic contraction for 7 s. Slack lower jaw, agonal convulsions and tail lifting commenced simultaneously immediately after the shot, with agonal convulsions lasting for only 1 s and tail lifting for 16 s. Tail fluttering, which was observed continuously prior to application of the shot, continued for 15 s post firearm discharge, whereas dorsal fin fluttering ended shortly after the shot and the left eye opened and remained open for 52 s following the shot. An audible terminal exhalation commenced 1.7 s after the shot and lasted 33.3 s. Relaxation of epaxial musculature occurred 24 s post-shot (Fig. 5).

###  Long-finned pilot whale PMCT examination: ballistics related

**Fig. 6:**
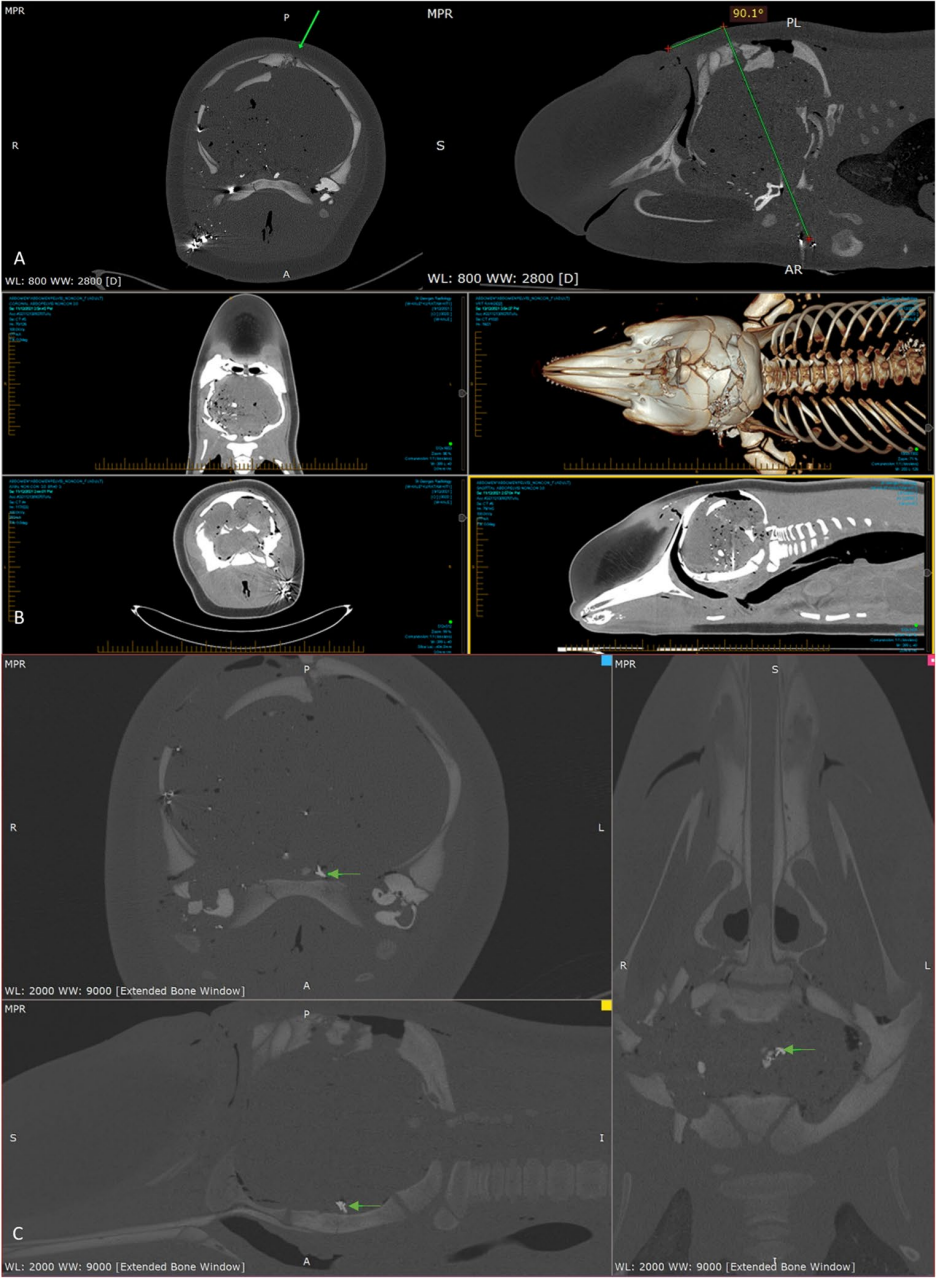
Multiplanar reconstruction (MPR) PMCT images of long-finned pilot whale (*Globicephala melas edwardii*) post-ballistics application: (**A**) angle of projectile trajectory (**B**) a soft tissue window (WL: 40; WW: 400) using Ineterad, InteleViewer and (**C**) the extended CT scale (WL: 2000; WW: 9000) using Medixant, RadiAnt DICOM Viewer. Note: Complete fracture of the occipital bone at projectile entry location, metallic and osseous bone fragments within brain parenchyma and metallic fragments marginally cranial to pectoral fin at projectile exit location. The green arrow depicts the evidence of metallic fragments that may have disrupted the rostral brainstem at the level of the pons

The single dorso-ventral shot penetrated the occipital blubber and muscle at the dorsal aspect of the frontal bone (Fig. 6), ca. 7.5 cm caudal to the blowhole. Internally the projectile trajectory was angled caudally at approximately 45° towards the right side. The projectile caused closed comminuted fractures and variably displaced bone fragments of the skull, including the maxillary, incisive, ethmoid, frontal, parietal, temporal, and occipital bones; fractures were most severe on the right side. Right-sided comminuted fractures of the occipital and paracondylar process of the basioccipital bone were present, with mild comminuted fracture extension to the left.

On impact, the projectile likely fractured with the occipital bone, resulting in trajectory deviation, with multifocal hyperattenuating foci compatible with metallic (projectile) fragments, throughout the right cerebral hemisphere. The right bulla was fractured with metal fragments around the right peribullar area; the right cochlear was markedly dorsally displaced into the brain parenchyma. The disruption of brain parenchyma likely caused flooding of the sinuses (likely haemorrhage), including the peribullary sinus. Multiple metal fragments were present along the exit trajectory with a narrow cluster within the muscles and subcutaneous tissues, 3 cm cranial to the right pectoral joint. This additional wounding outside the cranial cavity may have disrupted major blood vessels to the brain and heart. A moderate surface defect was present ventral to these metallic fragments, likely representing the distal (exit) trajectory (and the likely exit wound, though interpretation was hindered by beam hardening artefact from metal projectile fragments).

A metallic fragment (1 cm x 0.5 cm) was noted dorsal to the basisphenoid bone (Fig. 6, using the extended CT scale), possibly disrupting the rostral brainstem at the level of the pons. Scant projectile fragments were noted in the cervical segment of the spinal cord and at the level of T1/T2. Fractures were observed in both occipital condyles (Fig. 5) suggesting disruption to the brainstem was likely and near instantaneous insensibility would have occurred. Substantial subcutaneous and intracranial gas accumulations were observed, likely secondary to damage and fractures.

### Long-finned pilot whale PMCT examination: wider findings

The maternal dependence of the individual was confirmed by the presence of unfused epiphyseal plates in the pectoral fins, unfused cervical vertebral bodies and open ventral growth plates of the atlas (C1). Hyperattenuating materials in the oral cavity and blowhole sinuses were evident and were likely sand or sediments due to the live stranding of the individual. Similarly, hyperattenuating material was noted in the stomach and was likely ingested sand. There was some slight patchy increase in attenuation of the pulmonary parenchyma, though this may have been a post-mortem change.

###  Pygmy sperm whale: behavioural responses to application of ballistics

**Fig. 7:**
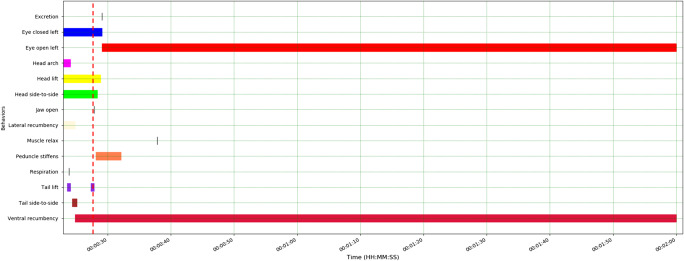
Behavioural events relating to the application of ballistics to the pygmy sperm whale (*Kogia breviceps*). The shot is indicated by the red dashed line. Total recording/observation duration: 211 s; ~97 s relevant to ballistics application shown here

Ballistics application was undertaken using a 0.357 calibre rifle with one 158 gr solid, jacketed round nose projectile. A single firearm discharge occurred with the marksperson standing in front of the animal’s head, with the firearm muzzle positioned approximately 15 cm above and in front of the animal’s head, angled at approximately 35° ventro-caudally to the right of the animal’s dorsal midline, aiming just caudal to the blowhole. The shot entered caudal and to the right of the animal’s blowhole. Following the shot there was a delay in the checking of insensibility criteria of ca. 2.5 min (154.9 s), but when tested both the palpebral and corneal reflexes were absent verifying animal insensibility. A total of 9 animal behavioural responses were characterised following firearm discharge (Fig. 7).

The lower jaw became slack and the peduncle stiffened immediately post-shot and the peduncle remained in this tonic contraction for 4 s. Urinary excretion and left eye opening were observed 1.5 s after the shot and relaxation of the epaxial musculature occurred 10.3 s post-application. Head lift, head side to side and tail lift, which were observed prior to application of the shot, continued for 1 s or less post firearm discharge.

###  Pygmy sperm whale PMCT examination: ballistics related

**Fig. 8:**
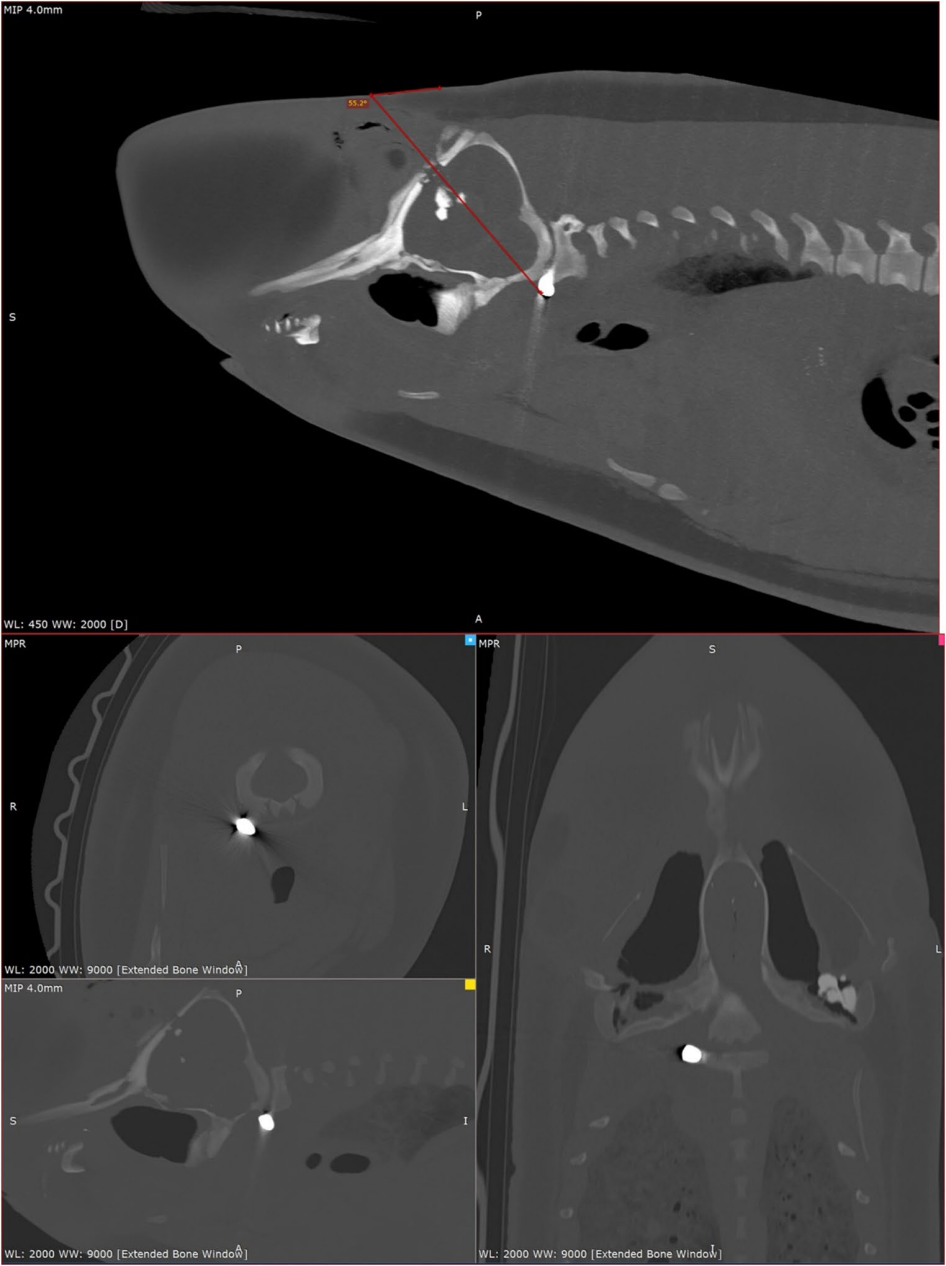
Multiplanar reconstruction (MPR) PMCT images of pygmy sperm whale (*Kogia breviceps*) post-ballistics application in the extended CT scale (WL: 2000; WW: 9000) using Medixant, RadiAnt DICOM Viewer. Note: Projectile lodged immediately right ventral to the atlanto-occipital joint

A single projectile entered via a dorsoventral, parasagittal approach at an approximately 55° angle internally (Fig. 8), slightly right-sided and caudal to the blowhole. A minimal superficial wound was noted at the entry site. A large (~ 8 cm in diameter) region of concentric emphysema and altered soft tissue density was present along the right side of the melon, compatible with cavitation and contusions along the projectile trajectory. There was penetration of the right premaxillary bone as the projectile entered the calvarium. A simple complete fracture of the body of the left maxilla was observed, likely secondary to impact (Fig. 8). Focal fractures at the ramus of the left mandible and right maxilla/premaxilla bone were observed. Bone attenuating fragments were noted caudal to the entry point through the brain parenchyma. These were compatible with fragments of the right premaxillary bone from the projectile entering the skull.

Evidence of considerable damage to the brain parenchyma in the right parietal lobe was observed, with bone fragments within the right ventricle. The terminal position of the projectile (HU = 3000+) was the right ventrolateral aspect of the atlas (C1), within the atlanto-occipital joint. The projectile remained smoothly marginated and whole. No metal fragmentation was noted. There were resultant fractures of the right occipital bone and ventral aspects of the occipital condyles (Fig. 8). The brainstem and spinal cord were directly physically damaged by the projectile but may not have been completely transected. This suggests that insensibility was likely instantaneous and no additional wounding outside the cranial cavity was observed.

### Pygmy sperm whale PMCT examination: wider findings

The presence of epiphyseal plates at the humerus, radius, and ulna of both flippers, alongside the presence of physeal endplates at various vertebral levels confirmed the animal’s early life-stage. Hyperattenuating foci compatible with sand were observed along the entire upper respiratory system, from nasal sinuses, nares and goosebeak; this was likely inhaled when the animal stranded alive. A mix of hypo- and hyperattenuating material was observed within the stomach, which was likely digesta and sand. A slight increase in pulmonary attenuation with ventral distribution, likely hypostatic post-mortem changes, was observed. Mild, diffuse pulmonary oedema predominantly showing a ground-glass attenuation pattern, with subpleural sparing, was suggestive of drowning and/or re-stranding impacts. The hypostatic congestion in both lungs was likely associated with the ventral recumbency of the animal during stranding and positional atelectasis but may also have reflected post-mortem changes. Mild pleural effusion was also noted. A moderate number of iso-attenuated nodules within blubber in the apex of the thorax (ventral at the axillary level), lumbar and caudal regions were noted, suggestive of intra-blubber parasitic infestation.

###  Killer whale: behavioural responses to ballistics application

**Fig. 9:**
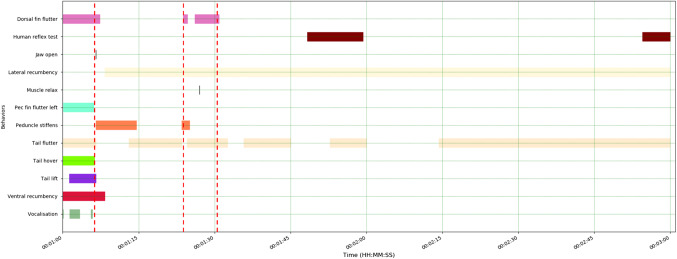
Behavioural events relating to the application of ballistics to the killer whale (*Orcinus orca*). The shots are indicated by the red dashed lines Total recording/observation duration: 277 s; 120 s relevant to ballistics application shown here

Ballistics was undertaken using a 0.308 calibre rifle with three 180 gr bonded soft point projectiles. The three pre-planned firearm discharges occurred with the marksperson standing in front of the animal, with the firearm muzzle positioned approximately 50 cm above and in front of the animal’s head, angled approximately 60° ventro-caudally along the animal’s dorsal midline, aiming through the blowhole. The shots entered through the centre of the animal’s blowhole. Criteria for verifying insensibility were assessed three times following the final application of ballistics, the first time at 18 s after the final shot (42 s after the initial firearm discharge); no responses were observed at any of the assessments, verifying animal insensibility. A total of six animal behavioural responses were characterised following shooting from the video footage (Fig. 9).

Following the first shot, ventral stiffening of the peduncle occurred immediately (< 1 s) and the peduncle remained in this tonic contraction for 8 s. Slack lower jaw commenced immediately after the shot. Tail fluttering, which was observed continuously prior to application of the shot, ceased when ballistics was applied but then began again 6.8 s post firearm discharge and continued for 10.5 s. Dorsal fin fluttering ended immediately after the first shot and only began again following the second shot.

The second shot occurred 17.5 s after the first and peduncle stiffening occurred immediately but lasted only 1.2 s. Dorsal and tail fin fluttering both began immediately following the second shot, while relaxation of the epaxial musculature occurred 3.2 s after the second shot (20.8 s post-initial shot; Fig. 9). Dorsal fin fluttering continued for 4.8 s, ceasing after the third shot, which was applied 6.7 s after the second shot (24.3 s post the initial shot), whereas tail fluttering continued intermittently over the following 98.7 s. At this point all animal behavioural responses ceased and no behaviours were observed for the final 118 s of video footage.

A visible heartbeat, assessed as rhythmic movement of the ventrum medial to the left pectoral fin, was observable for 88 s following the final shot at which point it ceased (112 s after the initial shot). Notably, the tail fluttering observed following the third shot ceased 5 s after presumed cardiac cessation.

### Killer whale PMCT examination: ballistics related

**Fig. 10:**
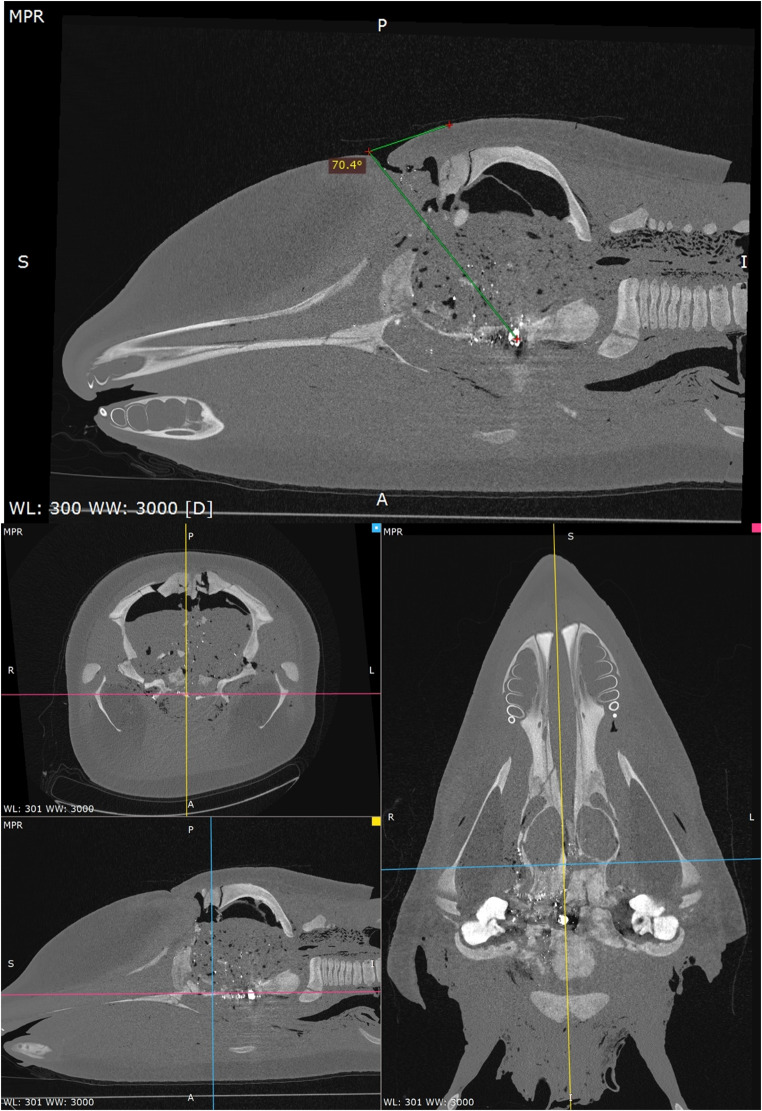
Multiplanar reconstruction (MPR) PMCT images of killer whale (*Orcinus orca*) post-ballistics application in a bone window (WL: 300; WW: 3000) using Medixant, RadiAnt DICOM Viewer. Note: main projectile fragments observed in the basisphenoid and basioccipital bones

The three projectiles entered the frontal region on a dorsoventral, sagittal trajectory, at approximately 70° internally, near the blowhole. There was penetration of the premaxillary, nasal, and frontal bones. Most of the projectiles fragmented into a narrow cluster of three parts. These terminated within the region of the highly comminuted, fractured, and variably displaced basisphenoid, right petrous temporal, and rostral basioccipital bones (Fig. 10). Regional emphysema was noted in the surrounding extra-calvaria structures, which may have been related to ballistics or post-mortem changes.

Fractures of the left zygomatic bone, vomer, right tympanic bulla, pterygoid, temporal, parietal, supraoccipital bones, and occipital condyles were also noted. Small, multifocal mineral attenuating foci (likely bone fragments) were observed tracking through the ballistic entry point and surrounding the main three projectile fragments. Few, pinpoint metal foci were also noted scattered throughout the brain parenchyma (Fig. 10). Both tympanic bulla and the nasal canal were filled with soft tissue attenuation, compatible with haemorrhage from ballistic trauma, though these may have been influenced by post-mortem changes.

The location of the three main projectile fragments, particularly within the basisphenoid and basioccipital bones, suggests direct and irreparable damage to the rostral brainstem. This likely resulted in near-instantaneous insensibility.

### Killer whale PMCT examination: wider findings

Only the entire head, seven cervical vertebrae, and two upper cranial thoracic vertebral bodies were available for PMCT scanning, therefore, additional findings throughout the body cavity could not be reported. The ventral growth plate of the C1 vertebral body was open and the cranial cervical vertebrae were unfused, evidencing the young age of the animal.

###  Killer whale: dissection

**Fig. 11:**
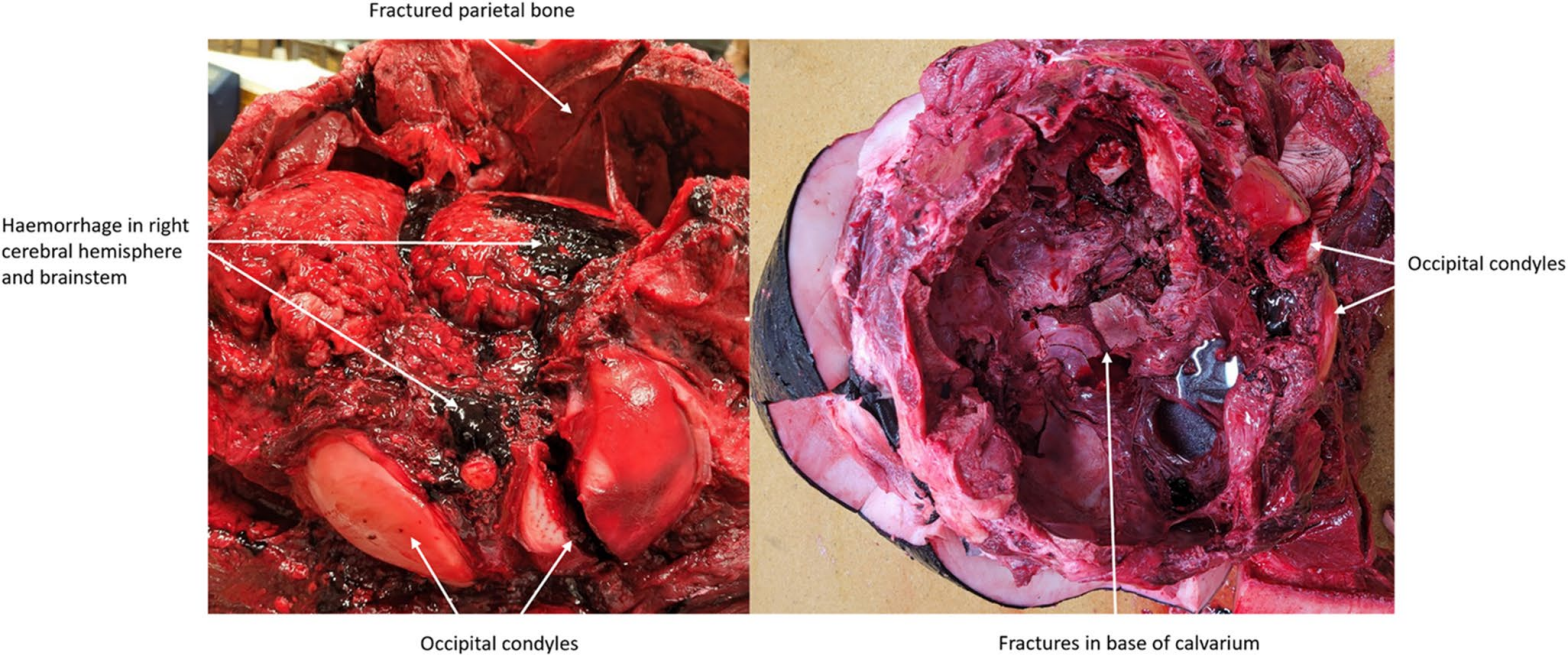
Opened cranium of the maternally-dependent killer whale (*Orcinus orca*) at necropsy evidencing damage caused by ballistics using a 0.308 calibre rifle with three 180 gr bonded soft point projectiles. Fractures in the right occipital condyle and parietal bones alongside haemorrhage in the right cerebral hemisphere and brainstem (left). Fractures in the base of the calvarium (right)

The PMCT findings were confirmed at dissection: the right occipital condyle had a complete fracture and fractures of the supraoccipital and parietal bones were also noted, particularly on the right (Fig. 11). Significant haemorrhage in the right cerebral hemisphere was observed and disruption of the cerebellum and brainstem, with surrounding haemorrhage, were noted. Severe fractures in the basioccipital and basisphenoid bones were found on removal of the brain (Fig. 11).

## Discussion and conclusions

To the best of our knowledge, this is the first study to combine analysis of cetacean behavioural responses following the application of ballistics with post-mortem CT findings to explore welfare implications and evaluate ballistics as a potentially humane killing method. Euthanasia of cetaceans is a vital option at stranding events where animals are severely welfare compromised or when rescue is not considered feasible (e.g., lack of resources and/or high safety risk for humans). The size, external features such as neonatal folds, and PMCT findings of open ventral growth plates in C1 and unfused epiphyseal plates in the pectoral fins confirmed the maternally-dependent age of all three odontocetes, thus supporting the decision to euthanise following the New Zealand Standard Operating Procedure (NZ SOP). However, it is critical that such procedures undergo robust scientific assessment to understand any potential welfare implications of the method itself and to ensure a humane death is achieved. Although limited by the number of animals assessed, these preliminary results indicate how welfare outcomes can be optimised through choice of firearm-projectile equipment and application of ballistics.

### Behavioural responses and PMCT findings relevant to ballistics application

A single shot was applied for ballistics killing of the maternally-dependent pilot whale and pygmy sperm whale, whilst three pre-planned shots were applied to the maternally-dependent killer whale. In all cases, brief peduncle stiffening (4–8 s), likely a tonic spasm, and irreversible slackening/opening of the lower jaw occurred almost immediately after the first/only shot, followed shortly afterwards by epaxial muscle relaxation (10–25 s after shooting). None of these behaviours were observed immediately prior to ballistics, indicating their direct consequence as a result of the procedure. Similar behaviour following application of ballistics to stranded cetaceans were reported previously (Boys et al. 2023) suggesting some consistent immediate behavioural responses among odontocetes when the brain is disrupted by ballistics. However, insensibility, which was confirmed in the field (via corneal and/or palpebral reflex testing) in two of the three cases here, was only verified 42 s after the first shot at the earliest (killer whale) and 2.5 min later in the pygmy sperm whale, hindering our ability to interpret the immediate behaviours in terms of loss of awareness. Furthermore, criteria to verify insensibility were not applied to the pilot whale at all, despite the requirement to do so in the NZ SOP (Boren 2012; Boys et al. 2022a).

Tail fluttering continued for 15 s post-ballistics in the pilot whale and intermittently over 98 s after the third shot in the killer whale. Additionally, dorsal fin fluttering was displayed by the killer whale between the second and third shot, though it ceased after the only shot applied to the pilot whale. Notably, in the killer whale, tail fluttering (and visible heartbeat) continued to occur long after insensibility was verified via loss of palpebral and corneal reflexes and after relaxation of the epaxial musculature. Indicating that these fasciculations/spasms can continue to occur after loss of brainstem reflexes. In both cases, the PMCT evidenced disruption to the brain parenchyma and rostral portion of the brainstem while the spinal cord was not directly affected. In contrast, no fluttering behaviours were displayed by the pygmy sperm whale which on PMCT presented direct damage to the rostral and caudal brainstem and spinal cord, with the projectile lodged at the atlanto-occipital joint. This indicates that these fluttering behaviours are likely to be involuntary clonic spasms related to continued spinal reflexes rather than being related to brainstem reflexes (Erasmus et al. 2010b; Kline et al. 2019). Therefore, we suggest that these fasciculations cannot be used as an indicator of consciousness or to confirm insensibility since it is possible that they would be absent in cases of paralysed, but conscious, animals.

Due to the possibility for animals to return to consciousness (Grandin 2002; Erasmus et al. 2010a), it is vital that they are carefully monitored until all movements have ceased and that testing of brainstem reflexes (e.g., absence of corneal reflex) which can verify insensibility are checked multiple times. Indeed, in this study the behavioural responses of the pilot whale post-shooting also included tail lifting, which began shortly after agonal convulsions and jaw slackening, and continued for about 15 s, at which time all active behaviour ceased before relaxation of epaxial musculature. Such movement is likely a prolonged tonic spasm in this case, given the disruption to the rostral brainstem and occipital condyles (Alonso-Farré et al. 2015; Cozzi et al. 2016). However, ongoing tail lifting and arching behaviour could be indicative of [return to] sensibility due to a functioning reticular formation, proprioception, and muscular tone (Grandin 2002; Cozzi et al. 2016; Leary et al. 2020), a concern that was noted in our previous study (Boys et al. 2023).

In contrast, no tail lifting movements were displayed by the pygmy sperm whale following shooting, and rapid (10 s) relaxation of musculature occurred. In contrast to the other animals, it is likely that both cerebral cortical control and spinal cord function were lost almost immediately and simultaneously in the pygmy sperm whale, indicated by the lack of any musculoskeletal movements, open jaw and urination followed relatively rapidly by musculature relaxation (Dawson et al. 2007, Erasmus et al. 2010). This is supported in the PMCT findings which indicate significant disruption to the brain parenchyma alongside direct physical damage to the brainstem, atlanto-occipital joint and spinal cord, suggesting minimal welfare impacts associated with this procedure (Parvizi and Damasio 2003; Thomson et al. 2013; Schwenk et al. 2016). While insensibility was confirmed via absence of the palpebral and corneal reflexes this only occurred long after all behaviour had ceased (2.5 min after shooting), hindering accurate assessment of time to insensibility and limiting our understanding of duration of any welfare impacts.

Conversely, the killer whale was confirmed unconscious 18 s after the third shot, which was 42 s after the initial shot. The cessation of an observable heartbeat suggested death of the animal occurred less than 2 min (112 s) after the initial shot was applied but it was clearly irreversibly insensible more than a minute before this. Based on behavioural responses, the killer whale likely lost cerebral cortical control immediately, followed by spinal cord dysfunction and brain death, indicated by the cessation of all musculoskeletal movements (Erasmus et al. 2010). Indeed, the PMCT and necropsy findings demonstrated fractures to the occipital condyles and basioccipital bones particularly, suggesting direct physical damage to the brainstem and near instant insensibility (Parvizi and Damasio 2003; Thomson et al. 2013; Schwenk et al. 2016). Therefore, welfare impacts of the procedure were likely minimal.

In this study we have taken a conservative approach to inferring instantaneous insensibility based on direct physical disruption to the brainstem; this is important since penetrating cranial ballistics injuries and brainstem lesions do not always result in permanent irreversible unconsciousness (Parvizi and Damasio 2003; Agrawal et al. 2008; Park et al. 2010). However, since the brain parenchyma is intolerant to stretching and given the enclosed nature of the cranium, the formation of a temporary cavity by a projectile and the resulting shockwave will likely cause profound effects on indirectly damaged regions, such as the brainstem and neural tissue (Courtney and Courtney 2007; AbdulAzeez et al. 2018).

Assessment of these functional impairments caused by such lesions in the brain is complex due to the difficulties in evaluating damage to the reticular formation, synapses, myelin sheath, neurites, and other neurological tissue which may play a role in consciousness (Parvizi and Damasio 2003; Narayana 2017). While these tissues could not be directly assessed in this study, we have described the pathological consequences of the ballistics methods applied and focussed our evaluation on the direct physical damage caused to the brainstem, which plays a critical role in the generation of consciousness through the reticular activating system (Parvizi and Damasio 2003; Grady et al. 2022; Taran et al. 2023) and determines the functioning of cardio-respiratory systems (Cozzi et al. 2016; DeNicola et al. 2019; Panneton and Gan 2020).

### Variation in ballistics application: PMCT findings

From the video footage, shot placement in all three animals appeared to be consistent with recommendations in the current national SOP for stranded cetaceans: all shots were applied through or slightly posterior to the blowhole and generally aimed towards the occipital condyles with the purpose of disrupting the brainstem. However, the results of the PMCT suggest variation in the actual trajectory and thus location and extent of direct damage to the targeted structures (Millar and Mills 2000; Dybdal et al. 2023). The type of projectile and angle of aim likely interacted with species-related differences in cranial anatomy to influence the observed damage.

To illustrate, the soft non-bonded projectile applied to the pilot whale fragmented on impact with the occipital bone, resulting in projectile deviation. The larger fragment continued into the right ear bone and exited cranial to the right pectoral fin, likely causing additional unplanned wounding outside the calvarium. Such additional wounding is particularly important to consider when animals may not have been rendered instantaneously insensible, as they may be aware of, and experiencing, negative affective states such as pain, discomfort, and fear. In this case, the fractured occipital condyles and minimal rostral brainstem disruption suggests the pilot whale would have been near-instantly insensible during the haemorrhaging in the peribullary sinus and other major thoracic blood vessels in the region of the pectoral fin (Cozzi et al. 2016). Whether such injuries could be related to the additional tail lifting behaviour observed remains unknown.

Similarly, the soft bonded projectile applied to the killer whale also fragmented, however, the bonded nature likely reduced fragmentation and minimised ricochet, resulting in a narrow cluster of major projectile fragments. Both bone and metallic projectile fragments were observed scattered throughout the brain parenchyma causing brain disruption and the major fragments terminated in the basisphenoid and basioccipital bones. This likely caused near instant insensibility which was also evidenced by only fluttering behaviours being displayed.

In contrast, the solid projectile applied to the pygmy sperm whale remained whole and on an appropriate trajectory. Bone fragments were distributed semi-linearly throughout the brain parenchyma from the entry site to the occipital bone, where both the brainstem and spinal cord were directly physically damaged, and the projectile remained lodged in proximity to the atlanto-occipital joint. This supports the idea of instantaneous insensibility alongside no additional behaviours being observed.

In cases where soft projectiles are utilised it is possible that direct disruption to the brainstem may not occur. This is due to the fact that soft projectiles expand and mushroom on impact to create a wider cavity with the aim to cause increased tissue disruption (Thomson et al. 2013). However, in species with thick soft-tissues and dense bone, such as cetaceans, they are likely to rapidly fragment on impact with bone, reducing penetration ability and lowering killing efficiency (Hollerman et al. 1990; Blackmore et al. 1995; IWC 2000; Øen and Knudsen 2007; Thomson et al. 2013; Hampton et al. 2014; Knox et al. 2018). Projectile fragmentation also increases the likelihood of intracranial ricochet causing deviations in trajectory (AbdulAzeez et al. 2018) and increasing the likelihood of additional lesions occurring outside the cranial cavity, as was observed in the pilot whale. In animals that are not rendered immediately insensible, such injuries present notable welfare concerns. For this reason, it is internationally recommended that non-deforming, solid projectiles are utilised for the killing of cetaceans to ensure sufficient penetration of the thick cranium (Duignan and Anthony 2000; Øen and Knudsen 2007; Hampton et al. 2014). Indeed, in this study the solid projectile remained on trajectory and terminated at the target point (atlanto-occipital joint) causing direct disruption to the brainstem and spinal cord and likely instantaneous insensibility with no or minimal welfare implications. However, it should be considered that an inappropriate angle of aim may result in a solid projectile not reaching the brainstem, leading to similar welfare concerns of only severe wounding rather than insensibility.

Notably, it is likely that the incomplete fusion of cranial sutures due to young age were an important factor that enabled increased disruption of brain tissue in the two cases utilising soft deforming projectiles. Although the projectiles deviated from the intended trajectory, the penetration of immature cranial bones and thin musculature by the expanding projectiles was still possible. In other cetacean species exhibiting relatively thin cranial bones and musculature (e.g., harbour porpoises *Phocoena phocoena* and Hector’s dolphins *Cephalorhynchus hectori*), such deforming projectiles may adequately penetrate the calvarium and reach the brainstem causing direct disruption or cause sufficient damage in the brain parenchyma to cause indirect disruption to the brainstem. However, if such projectiles were utilised on a mature animal with fully calcified, fused cranial bones and dense musculature (e.g., bottlenose dolphin *Tursiops truncatus* or pilot whale), it is possible that only serious wounding and inadequate disruption of brain tissue to cause rapid loss of consciousness may occur, leading to severe welfare compromise (Gascho et al. 2022).

In addition to projectile type, the angle of aim towards the anatomical landmark is likely to influence the accuracy of direct disruption to the brainstem. For example, based on the limited findings here, it seems that an obtuse angle (i.e., 35°), rather than acute angle (i.e., 70°), improved the accuracy of the projected trajectory towards the brainstem. This aligns with Hampton et al. (2014) who reported the use of an angle aim of 45° to disrupt the brain of cetacean carcasses using ballistics. However, species-specific differences will likely further impact outcomes, especially with the asymmetry noted in *Kogia* cranial anatomy (Thornton et al. 2015, Huggenberger et al. 2017). Differences in cranial morphology of similar sized odontocetes, such as beaked whales (Gol’din 2014; Roston and Roth 2019), would further exacerbate the effects of species variance on projected trajectory and effective brain disruption. Therefore, improved understanding of and detailed guidance on species-specific targets are needed to ensure optimal animal welfare outcomes.

Given the young age of the animals presented here, the cranial morphology, thin musculature and relevant cranial disruption are not representative of the likely outcomes across differing maturity stages or other species. Accordingly, further research should examine ballistics killing of various cetacean species and at differing stages of physical/osteological maturity, to better assess the relative efficacy and humaneness of the method. This research should include the evaluation of the types and prevalence of additional unintentional lesions that occur.

### Recommendations

At the time of ballistics application to these animals, the recommendation in the NZ SOP (Boren 2012) for stranded cetacean euthanasia was to use expanding/soft projectiles (Boys et al. 2022a), with such projectiles reported used on various cetacean species in New Zealand (Boys et al. 2021, 2023). This differs to best practice SOPs across Australia which typically recommend only solid, non-deforming projectiles (Hampton et al. 2014; Boys et al. 2022a). The recommendation of soft projectiles in the NZ SOP may be due in part to human safety concerns, as it is thought that non-deforming, solid projectiles are more likely to pass through an animal and then ricochet. However, in this study, it was the soft non-bonded projectile rather than the solid projectile that was observed to exit the cranial cavity. Further evaluation of ballistics euthanasia for cetaceans should be conducted to better understand and mitigate these risks.

Despite being mandated in the SOP, verification of insensibility was only undertaken at the appropriate time in one of the animals. It is critical that criteria for verifying insensibility are systematically and *immediately* assessed following the application of a killing method. Only in this way can we truly evaluate the welfare implications of the procedure. Notably, in this study multiple shots were planned for only one animal, despite this being the recommendation in the NZ SOP and wider international guidelines (Boren 2012; Hampton et al. 2014). These two factors suggest that regular practical training and open discussion of the SOP among those working in wildlife management would contribute to improved animal welfare outcomes. With the advent of technologies such as 3D printing, it would be particularly beneficial to develop anatomically correct training models of various cetacean species and sizes (Yuen et al. 2017, Kot et al. 2020b). These could be used in regular training of staff to ensure that the aim applied is correctly targeting the brainstem in the species (e.g., Dybdal et al. 2023), optimising both animal welfare and human safety.

Notably, a lack of training has been highlighted previously as a key concern for those involved in cetacean euthanasia globally (Barco et al. 2016; Stringfellow et al. 2022). Despite international guidelines existing, there is a significant lack of data on euthanasia procedures applied at stranding events, particularly in terms of the projectiles employed and whether, and how, insensibility is verified (Boys et al. 2021). This leads to limited knowledge regarding the welfare implications of euthanasia procedures being applied globally and highlights a vital need for improved training and reporting standards at an international scale.

Due to the challenges with inferring welfare impacts from PMCT images of ballistics-related damage discussed above, additional imaging examinations, such as post-mortem magnetic resonance imaging (PMMRI) and post-mortem angiography, and detailed dissection and histopathology on euthanised animals should be undertaken where possible to better evaluate the amount and specific regions of tissue disruption (Narayana 2017; Kot et al. 2020a, b; Tsui et al. 2020). This would also provide more robust data to inform assessment of welfare implications, as has been undertaken on humans (Oehmichen et al. 2003; Thali et al. 2003). The combination of imaging and detailed dissection would be particularly valuable for understanding damage to the reticular formation which would likely affect animal awareness/insensibility, but for which assessment is complex (Parvizi and Damasio 2003; Cozzi et al. 2016; Faraguna et al. 2019). The value of imaging technologies has been highlighted here in terms of improving cetacean euthanasia, however, we suggest that the application of such technology to assess animal welfare implications in other contexts and species, such as animal cruelty cases, would also be valuable and should be considered (e.g., Park et al. 2010). Such examinations should be performed by experienced and/or certified radiological clinicians or radiologists to ensure appropriate interpretation and reporting (Tsui et al. 2020).

Finally, it is also important that various options and their risks at each live stranding event are carefully considered, noting that euthanasia may not be immediately possible (even if required) if the animal is fractious. In the cases reported here, the animals were sufficiently debilitated that behaviour did not pose a threat to human safety and/or thwart the option of euthanasia. Human safety considerations are of paramount concern and the area surrounding an animal must be cleared of personnel prior to utilising ballistics so as to mitigate the risk of gunshot associated with a perforating wound and ricochet projectile.

## Conclusion

Our results suggest that in small, immature cetaceans with unfused cranial sutures, a relatively rapid, humane death can be achieved via ballistics. However, our findings have also highlighted several potential concerns that must be considered to ensure effective euthanasia, including not only physical maturity but also species anatomy, angle of aim, and projectile trajectory. Importantly, the type of projectile applied must be appropriate for the species and maturity of the animal to cause penetration of the cranial cavity with minimal deviation, ensuring that the brainstem is disrupted, and instantaneous or at least very rapid insensibility is achieved. Our case studies also emphasise the importance of following all aspects of euthanasia SOPs; it is imperative that insensibility and/or death are verified *immediately* post-application of the euthanasia method and that detailed, accurate reporting is consistently undertaken to allow for the assessment and improvement of procedures.

## Data Availability

The datasets generated during and/or analysed during the current study are available in non-identifiable format from the corresponding author on reasonable request.

## References

[CR1] AbdulAzeez MM, Dolachee AA, Huber P-Z et al (2018) Intracranial ricocheted-bullet injuries: an overview and illustrative case. J Acute Dis 7. 10.4103/2221-6189.244165

[CR2] Agrawal A, Pratap A, Rauniar R et al (2008) Intracranial ricocheting of bullet from anterior clinoid process. J Nepal Med Assoc 47. 10.31729/jnma.325 19079382

[CR3] Alonso-Farré JM, Gonzalo-Orden M, Barreiro-Vázquez JD et al (2015) Cross-sectional anatomy, computed tomography and magnetic resonance imaging of the Head of Common Dolphin (*Delphinus delphis*) and Striped Dolphin (*Stenella coeruleoalba*). Anat Histol Embryol 44:13–21. 10.1111/ahe.12103 24527804 10.1111/ahe.12103

[CR4] Arbelo M, de Los Monteros AE, Herráez P et al (2013) Pathology and causes of death of stranded cetaceans in the Canary Islands (1999–2005). Dis Aquat Organ 103:87–99. 10.3354/dao02558 23548359 10.3354/dao02558

[CR5] Barco SG, Walton WJ, Harms CA et al (2016) Collaborative development of recommendations for euthanasia of stranded cetaceans. US NOAA, Silverspring, Maryland, USA

[CR6] Beausoleil NJ, Fisher P, Littin KE et al (2016) A systematic approach to evaluating and ranking the relative animal welfare impacts of wildlife control methods: poisons used for lethal control of brushtail possums (*Trichosurus vulpecula*) in New Zealand. Wildl Res 43:553–565. 10.1071/WR16041

[CR7] Betty E (2019) Life history of the long-finned pilot whale (*Globicephala melas edwardii*); insights from strandings on the New Zealand coast. PhD thesis, Auckland University of Technology, New Zealand

[CR8] Bischoff K, Jaeger R, Ebel JG (2011) An unusual case of relay pentobarbital toxicosis in a dog. J Med Toxicol 7:236–239. 10.1007/s13181-011-0160-8 21660622 10.1007/s13181-011-0160-8 PMC3550208

[CR9] Blackmore DK, Madie P, Bowling MC et al (1995) The use of a shotgun for euthanasia of stranded cetaceans. New Zeal Vet J 43:158–159. 10.1080/00480169.1995.35878 10.1080/00480169.1995.35878 16031838

[CR10] Boren L (2012) Area operational plan for marine mammal incidents. Guidelines. Department of Conservation, Wellington, New Zealand

[CR11] Boys RM, Beausoleil NJ, Betty EL, Stockin KA (2021) Deathly silent: exploring the global lack of data relating to stranded cetacean euthanasia. Animals 11:1460. 10.3390/ani11051460 34069749 10.3390/ani11051460 PMC8161157

[CR12] Boys RM, Beausoleil NJ, Betty EL, Stockin KA (2022a) When and how to say goodbye: an analysis of standard operating procedures that guide end-of-life decision-making for stranded cetaceans in Australasia. Mar Pol 138:104949. 10.1016/j.marpol.2021.104949

[CR13] Boys RM, Beausoleil NJ, Pawley MDM et al (2022b) Evaluating potential cetacean welfare indicators from video of live stranded long-finned pilot whales (*Globicephala melas edwardii*). Animals 12. 10.3390/ani12141861 10.3390/ani12141861 PMC9312325 35883407

[CR14] Boys RM, Beausoleil NJ, Hunter S et al (2023) Assessing animal welfare during a stranding of pygmy killer whales (*Feresa attenuata*). Mar Mamm Sci. 10.1111/mms.13029

[CR15] Câmara N, Sierra E, Fernández A et al (2020) Capture myopathy and stress cardiomyopathy in a live-stranded Risso’s Dolphin (Grampus griseus) in Rehabilitation. Anim (Basel) 10:220. 10.3390/ani10020220 10.3390/ani10020220 PMC7070958 32013196

[CR16] Courtney A, Courtney M (2007) Links between traumatic brain injury and ballistic pressure waves originating in the thoracic cavity and extremities. Brain Inj 21:657–662. 10.1080/02699050701481571 17653939 10.1080/02699050701481571

[CR17] Cozzi B, Oelschläger H, Huggenberger S (2016) Anatomy of Dolphins. Insights into body structure and function. Academic, London, UK

[CR18] DeNicola AJ, Miller DS, DeNicola VL et al (2019) Assessment of humaneness using gunshot targeting the brain and cervical spine for cervid depopulation under field conditions. PLoS ONE 14:e0213200. 10.1371/journal.pone.0213200 30818392 10.1371/journal.pone.0213200 PMC6395039

[CR19] Diaz-Delgado J, Fernandez A, Sierra E et al (2018) Pathologic findings and causes of death of stranded cetaceans in the Canary Islands (2006–2012). PLoS ONE 13:e0204444. 10.1371/journal.pone.0204444 30289951 10.1371/journal.pone.0204444 PMC6173391

[CR20] Duignan PJ, Anthony D (2000) Marine mammal strandings - a vet’s role. Kokako 7(1):3–5

[CR21] Dybdal N, Horgan M, Costa L et al (2023) Equine gunshot euthanasia: creation of a 3D-Printed model with integrated sensors for training. Animals 13. 10.3390/ani13162566 10.3390/ani13162566 PMC10452018 37627357

[CR22] Erasmus MA, Lawlis P, Duncan IJH, Widowski TM (2010a) Using time to insensibility and estimated time of death to evaluate a nonpenetrating captive bolt, cervical dislocation, and blunt trauma for on-farm killing of turkeys. Poult Sci 89:1345–1354. 10.3382/ps.2009-00445 20548061 10.3382/ps.2009-00445

[CR23] Erasmus MA, Turner PV, Widowski TM (2010b) Measures of insensibility used to determine effective stunning and killing of poultry. J Appl Poult Res 19:288–298. 10.3382/japr.2009-00103

[CR24] Faraguna U, Ferrucci M, Giorgi FS, Fornai F (2019) Editorial: the functional anatomy of the reticular formation. Front Neuroanat 13. 10.3389/fnana.2019.00055 10.3389/fnana.2019.00055 PMC6548970 31191262

[CR25] Fraga-Manteiga E, Shaw DJ, Dennison S et al (2014) An optimized computed tomography protocol for metallic gunshot head trauma in a seal model. Vet Radiol Ultrasound 55:393–398. 10.1111/vru.12146 25184173 10.1111/vru.12146

[CR26] Friard O, Gamba M (2016) BORIS: a free, versatile open-source event-logging software for video/audio coding and live observations. Methods Ecol Evol 7:1325–1330

[CR27] Gales N, Leaper R, Papastavrou V (2008) Is Japan’s whaling humane? Mar Pol 32:408–412. 10.1016/j.marpol.2007.08.004

[CR28] Gascho D, Stephan R, Bauer C et al (2022) BigBovid- evaluation of a newly developed 9 mm bullet-shooting stunner for adequate stunning of heavy cattle. Front Vet Sci 9. 10.3389/fvets.2022.949198 10.3389/fvets.2022.949198 PMC9363664 35968016

[CR29] Gol’din P (2014) Antlers inside’: are the skull structures of beaked whales (Cetacea: Ziphiidae) used for echoic imaging and visual display? Biol J Linn Soc 113:510–515. 10.1111/bij.12337

[CR30] Grady FS, Boes AD, Geerling JC (2022) A century searching for the neurons necessary for wakefulness. Front Neurosci 16. 10.3389/fnins.2022.930514 10.3389/fnins.2022.930514 PMC9344068 35928009

[CR31] Grandin T (2002) Return-to-sensibility problems after penetrating captive bolt stunning of cattle in commercial beef slaughter plants. J Am Vet Med Assoc 221:1258–1261 12418689 10.2460/javma.2002.221.1258

[CR32] Greer L, Whaley J, Rowles T (2001) Euthanasia. In: Gulland LD and F (ed) CRC Handbook of marine mammal medicine, 2nd edn. CRC, Florida, USA, pp 729–738

[CR33] Hampton J, Mawson P, Coughran D, Vitali S (2014) Validation of the use of firearms for euthanising stranded cetaceans. J Cetac Res Manage 14:117–123

[CR34] Harms CA, Greer L, Whaley J, Rowles TK (2018) Euthanasia. CRC handbook of marine mammal medicine, 3rd edn. CRC, Boca Raton, USA, pp 675–691

[CR35] Herráez P, de los Espinosa A, Fernandez A et al (2013) Capture myopathy in live-stranded cetaceans. Vet J 196:181–188. 10.1016/j.tvjl.2012.09.021 23146174 10.1016/j.tvjl.2012.09.021

[CR36] Hollerman JJ, Fackler ML, Coldwell DM, Ben-Menachem Y (1990) Gunshot wounds: 1. Bullets, ballistics, and mechanisms of injury. Am J Roentgenol 155:685–690. 10.2214/ajr.155.4.2119095 2119095 10.2214/ajr.155.4.2119095

[CR37] IJsseldijk LL, Brownlow AC, Mazzariol S (2019) Best practice on cetacean post mortem investigation and tissue sampling. ACCOBAMS and ASCOBANS

[CR38] IWC (2000) Report of the Working Group on Whale Killing Methods and Associated Welfare issues. 30 June 2000, Adelaide. International Whaling Commission, Adelaide, Australia

[CR39] IWC (2014) Report of the IWC Workshop on Euthanasia Protocols to optimize Welfare concerns for stranded cetaceans. International Whaling Commission, Cambridge, UK

[CR40] Kline HC, Wagner DR, Edwards-Callaway LN et al (2019) Effect of captive bolt gun length on brain trauma and post-stunning Hind limb activity in finished cattle *Bos taurus*. Meat Sci 155:69–73. 10.1016/j.meatsci.2019.05.004 31082781 10.1016/j.meatsci.2019.05.004

[CR41] Knox AJ, Lardner B, Yackel Adams A, Reed RN (2018) Evaluating airsoft electric guns for control of invasive brown treesnakes. Wildl Soc Bull 42:534–539. 10.1002/wsb.909

[CR42] Knudsen S (2005) A review of the criteria used to assess insensibility and death in hunted whales compared to other species. Vet J 169:42–59 15683763 10.1016/j.tvjl.2004.02.007

[CR43] Kot BCW, Chan DKP, Chung TYT, Tsui HCL (2020a) Image rendering techniques in postmortem computed tomography: evaluation of biological health and profile in stranded cetaceans. J Vis Exp 163. 10.3791/61701 10.3791/61701 33044465

[CR44] Kot BCW, Tsui HCL, Chung TYT, Lau APY (2020b) Postmortem neuroimaging of cetacean brains using computed tomography and magnetic resonance imaging. Front Mar Sci 7:775. 10.3389/fmars.2020.544037

[CR45] Kot BCW, Ho HHN, Martelli P et al (2022) An Indo-Pacific humpback dolphin (*Sousa chinensis*) severely injured by vessel collision: live rescue at sea, clinical care, and postmortem examination using a virtopsy-integrated approach. BMC Vet Res 18:417. 10.1186/s12917-022-03511-1 36435769 10.1186/s12917-022-03511-1 PMC9701411

[CR46] Leary S, Underwood W, Anthony R et al (2020) AVMA guidelines for the Euthanasia of animals: 2020 edition. J Am Vet Med Assoc, Schaumburg, Illinois

[CR47] Link TM, Berning W, Scherf S et al (2000) CT of metal implants: reduction of Artifacts using an extended CT scale technique. J Comput Assist Tomogr 24 10.1097/00004728-200001000-00029 10667677

[CR48] Littin KE, Mellor DJ, Warburton B, Eason CT (2004) Animal welfare and ethical issues relevant to the humane control of vertebrate pests. New Zeal Vet J 52:1–10. 10.1080/00480169.2004.36384 10.1080/00480169.2004.36384 15768076

[CR49] Lund JR, Ketover HR, Hetzel S et al (2021) Computed tomographic assessment of brain tissue disruption and skull damage in equine cadaveric heads caused by various firearm-ammunition combinations applied as potential gunshot methods for euthanasia of horses. Am J Vet Res 82:28–38. 10.2460/ajvr.82.1.28 33369492 10.2460/ajvr.82.1.28

[CR50] Millar GI, Mills DS (2000) Observations on the trajectory of the bullet in 15 horses euthanased by free bullet. Vet Rec 146:754–757. 10.1136/vr.146.26.754 10909908 10.1136/vr.146.26.754

[CR51] Narayana PA (2017) White matter changes in patients with mild traumatic brain injury: MRI perspective. Concussion 2:CNC35. 10.2217/cnc-2016-0028 30202576 10.2217/cnc-2016-0028 PMC6093760

[CR52] O’Rourke K (2002) Euthanatized animals can poison wildlife: veterinarians receive fines. J Am Vet Med Assoc 220:146–147 12126115

[CR53] Oehmichen M, Gehl H-B, Meissner C et al (2003) Forensic pathological aspects of postmortem imaging of gunshot injury to the head: documentation and biometric data. Acta Neuropathol 105:570–580. 10.1007/s00401-003-0683-4 12664319 10.1007/s00401-003-0683-4

[CR54] Øen E, Knudsen S (2007) Euthanasia of whales: the effect of.375 and. 485 calibre round nosed, full metal-jacketed rifle bullets on the central nervous system of the common minke whale. J Cet Res Manage 9:81–88

[CR55] Panneton WM, Gan Q (2020) The mammalian diving response: inroads to its neural control. Front Neurosci 14. 10.3389/fnins.2020.00524 10.3389/fnins.2020.00524 PMC7290049 32581683

[CR56] Park S, Park J, Kim JM et al (2010) Penetrating cranial injury due to gunshot in a dog: a case report. Vet Med 55:253–257

[CR57] Parvizi J, Damasio AR (2003) Neuroanatomical correlates of brainstem coma. Brain 126:1524–1536. 10.1093/brain/awg166 12805123 10.1093/brain/awg166

[CR58] Plön S, Best PB, Duignan P et al (2023) Population structure of pygmy (*Kogia breviceps*) and dwarf (*Kogia sima*) sperm whales in the Southern Hemisphere may reflect foraging ecology and dispersal patterns. In: Plön S (ed) Advances in marine biology. Academic, pp 85–114 10.1016/bs.amb.2023.09.001 37980130

[CR59] Roston RA, Roth VL (2019) Cetacean skull telescoping brings evolution of cranial sutures into focus. Anat Rec 302:1055–1073. 10.1002/ar.24079 10.1002/ar.24079 PMC9324554 30737886

[CR60] Schiffer K, Retz S, Richter U et al (2014) Assessment of key parameters for gunshot used on cattle: a pilot study on shot placement and effects of diverse ammunition on isolated cattle heads. Anim Welf 23:479–489. 10.7120/09627286.23.4.479

[CR61] Schwenk BK, Lechner I, Ross SG et al (2016) Magnetic resonance imaging and computer tomography of brain lesions in water buffaloes and cattle stunned with handguns or captive bolts. Meat Sci 113:35–40. 10.1016/j.meatsci.2015.11.010 26610289 10.1016/j.meatsci.2015.11.010

[CR62] Stringfellow H, Butterworth A, Simmonds M (2022) An analysis of the approaches taken around the world to whale euthanasia. Anim Welf 31:113–123. 10.7120/09627286.31.1.010

[CR63] Taran S, Gros P, Gofton T et al (2023) The reticular activating system: a narrative review of discovery, evolving understanding, and relevance to current formulations of brain death. Can J Anaesth 70:788–795. 10.1007/s12630-023-02421-6 37155119 10.1007/s12630-023-02421-6 PMC10203024

[CR64] Thali MJ, Yen K, Vock P et al (2003) Image-guided virtual autopsy findings of gunshot victims performed with multi-slice computed tomography (MSCT) and magnetic resonance imaging (MRI) and subsequent correlation between radiology and autopsy findings. Forensic Sci Int 138:8–16. 10.1016/S0379-0738(03)00225-1 14642714 10.1016/s0379-0738(03)00225-1

[CR65] Thomson DU, Wileman BW, Rezac J et al (2013) Computed tomographic evaluation to determine efficacy of euthanasia of yearling feedlot cattle by use of various firearm-ammunition combinations. Am J Vet Res 74:1385–1391. 10.2460/ajvr.74.11.1385 24168302 10.2460/ajvr.74.11.1385

[CR66] Tsui HCL, Kot BCW, Chung TYT, Chan DKP (2020) Virtopsy as a revolutionary tool for cetacean stranding programs: implementation and management. Front Mar Sci 7. 10.3389/fmars.2020.542015

